# Effects of Dog-Assisted Education on Physical and Communicative Skills in Children with Severe and Multiple Disabilities: A Pilot Study

**DOI:** 10.3390/ani11061741

**Published:** 2021-06-10

**Authors:** Luis Lucio Lobato Rincón, Beatriz Rivera Martín, María Ángeles Medina Sánchez, Santos Villafaina, Eugenio Merellano-Navarro, Daniel Collado-Mateo

**Affiliations:** 1 Animal-Assisted Intervention Office, King Juan Carlos University, Móstoles, 28933 Madrid, Spain; luislucio.lobato@urjc.es (L.L.L.R.); danicolladom@gmail.com (D.C.-M.); 2 Department of Psychology, King Juan Carlos University, Alcorcón, 28922 Madrid, Spain; 3 Department of Communication and Sociology Sciences, King Juan Carlos University, Fuenlabrada, 28943 Madrid, Spain; 4 Department of Statistics and Data Science, Universidad Complutense, 28040 Madrid, Spain; amedina@estad.ucm.es; 5 Physical Activity and Quality of Life Research Group (AFYCAV), Faculty of Sport Sciences, University of Extremadura, 10003 Caceres, Spain; 6 Grupo de Investigacion EFISAL, Universidad Autónoma de Chile, 3460000 Talca, Chile; emerellano@gmail.com; 7 Centre for Sport Studies, Rey Juan Carlos University, Fuenlabrada, 28943 Madrid, Spain

**Keywords:** animal-assisted intervention, animal-assisted educational program, dogs, disability

## Abstract

**Simple Summary:**

Animal-assisted interventions have benefits in different populations, such as children with cerebral palsy or autism spectrum disorder. In this regard, human–animal interaction leads to different physical, cognitive, and emotional benefits in the child. Therefore, this pilot study aimed to evaluate the effects of a dog-assisted education program on the postural, oculomotor, linguistic, and autonomy dimensions in children affected by severe and multiple disabilities. A total of 14 children aged 3–12 years and affected by intellectual and physical disabilities participated in 12 sessions of the dog-assisted program where participants had to play with dogs. Children who participated in the study improved their postural control, eye-motor coordination, expression of sensations and feelings, spontaneous interaction, autonomy, and confidence.

**Abstract:**

Animal-assisted interventions have shown promising benefits in different populations such as children with cerebral palsy or autism spectrum disorder. Human–animal interaction leads to different physical, cognitive, and emotional benefits in the child. The aim of the current pilot study was to evaluate the effects of a dog-assisted education program on the postural, oculomotor, linguistic and autonomy dimensions in children affected by severe and multiple disabilities. Fourteen children aged 3–12 years and affected by intellectual and physical disabilities participated in a dog-assisted program consisted of 12 sessions. The intervention involved different types of activities, exercises, and games with the dogs. A strict protocol to ensure animal wellbeing and avoid any type of stress or fatigue was followed. Children who participated in the study improved their postural control, eye-motor coordination, expression of sensations and feelings, spontaneous interaction, autonomy, and confidence. However, these results must be taken with caution due to the lack of a control group and the heterogeneity of the participants.

## 1. Introduction

Animal-Assisted Interventions (AAI) can be defined as structured interventions that include animals to achieve a specific goal, such as improving physical or mental health (Animal-Assisted Therapy or AAT), enhancing cognitive function or social skills (Animal-Assisted Education or AAE), or simply increasing motivation and with recreational purposes (Animal-Assisted Activities or AAA) [1]. The scientific basis derives from the benefits of human–animal interaction, which has been previously described and is based on the facilitation of the social interaction and the presence of a source of non-judgment support [2,3]. This human–animal interaction is complex due to the involvement of two complex organisms (a human and an animal) interacting dynamically and being a source of large variability [4].

We can find many examples about the benefits of AAT in physical and psychological health [5,6] in patients with multiple sclerosis [7], older adults [8], autism disorder [9,10], children with attention-deficit/hyperactivity disorder [11], cerebral palsy [12], chronic pain [13], dementia [14], or cancer [15]. Furthermore, AAE have shown benefits on the development of social and communicational skills [10,14], prisoner reintegration [16], reading skills [17], motivation [18], enjoyment [19], empathy [20], or happiness [21].

Based on the large list of potential benefits, AAI could be a useful strategy to improve overall health while, at the same time, the patient is having an enjoyable time. Although the reported educational benefits (empathy or social skills, for example) are useful for almost everyone, they may be especially appropriate for children. In this regard, AAE may be considered a useful tool for children with developmental disorders and multiple disabilities. In this case, the animal is a source of continuous stimuli that leads to different physical, cognitive, and emotional reactions from the child, which, consequently, promotes the improvement of useful skills and behaviors. Among others, studies have shown improvements in social behavior, verbal communication, ability to follow instructions, and eye contact [22,23,24]. A recent review showed an improvement in social interaction in children with autism spectrum disorder in 22 studies after an AAI [25], in addition to an increase in positive emotions with this same population. Furthermore, another review also pointed out that AAI is useful to enhance gross motor function, social functioning, upper limb dexterity, balance, and mental health in patients with motor, neurological, or developmental disorders who need neurorehabilitation [26].

In sum, previous research has shown that AAIs for children lead to a list of benefits in different levels, including physical development (improvement in gross motor function, balance, or dexterity), enhancement of social and communicative skills, as well as cognitive and emotional development. These benefits may be achieved through an enjoyable and motivating program, which is especially important in children and, concretely, in those kids with severe and multiple disabilities that are usually receiving many treatments from the health service.

Therefore, the present pilot study aimed to evaluate the effects of the AAE program “One dog one happy child” on the psychomotor, social, and cognitive development (by enhancing the postural, oculomotor, linguistic, and autonomy dimensions) in children affected by severe and multiple disabilities. Given the multidimensional benefits of AAI in children and the close connection between motor dimensions and emotional wellbeing in childhood [27,28], benefits in the motor and affective sphere were expected after the application of the program.

## 2. Materials and Methods

### 2.1. Design

This is a quantitative observational pilot study. It included a baseline observation and within-session observations during each of the AAE sessions. Procedures were approved by the bioethical research committee of the hospital (number 19/194).

It is important to note that the present study had to be prematurely canceled after 12 sessions due to the State of Alarm decreed in Spain on 15 March 2020 and the health circumstances due to the SARS-CoV-2 pandemic.

### 2.2. Participants

This pilot study included a total of 14 children, aged 46 to 150 months, with different physical and intellectual disabilities. They were distributed according to their functional capacities in 3 subgroups of 4 or 5 children in order to increase the homogeneity of the groups. Each subgroup attended the AAE sessions twice a month.

Participants were recruited by the Ana Carolina Diez Mahou Foundation. This was a non-profit organization with the aim of improving the quality of life of children with neuromuscular disorders. To participate in the study, the inclusion criteria were: (a) more than 3 years of age; and (b) functional autonomy (be able to move autonomously). Exclusion criteria were established as: (a) The existence of severe medical conditions that makes the intervention unsuitable (such as the use of survival devices like ventilators); (b) the existence of allergies that could affect the child’s health; and (c) not having the written informed consent signed by their parents.

### 2.3. Data Collection Techniques and Measurements

Due to children included in the current study were repeatedly evaluated and tested by the medical staff, while their parents were usually stressed and affected by the health and the needs of their children, the measures aimed to minimize the impact on their daily living. Therefore, the selected tool was an Observation Scale used to analyze the children’s behavior during the sessions. This scale was designed by the research team with the opinion and collaboration of a pool of experts in the fields of education, psychology, sport sciences, communication, and occupational therapy. The scale was very simple, and the observers had to value the frequency a behavior appeared during sessions from 1 (the desired behavior never occurs) to 5 (the desired behavior always occurs). It was recorded in each session by a member of the research team without interrupting the normal rhythm of the session. There were 2 observers throughout the intervention period: HI and LJ.

The scale comprised the following 10 items that could be classified in the following sub-scales:

1. Postural control: Level of management of the body’s ability to maintain correct alignment of its body axis, facilitating the work of all joints and body segments, and coordinating the various muscular tensions to balance posture.
2. Oculomotor coordination: Level of coordination of body movements with vision. This sub-scale is comprised of 3 items: (a) One-handed ball grip and throwing, (b) two-handed ball grip and throwing, and (c) reception of an object in different situations.
3. Language and communication: Level of expressive ability ranging from simple vocalizations to the expression of thoughts and feelings through words. In this case, 4 items are measured: expression of sensations, expression of feelings, spontaneous interaction, and verbal communication.
4. Autonomy: Degree of independence and initiative when performing everyday behaviors and problem-solving. This dimension was comprised of 2 items: autonomy (the child conducts the activities of the session without help) and confidence (the child shows confidence in the activities, knowing what they have to do and how).

The characteristics of the program conducted in this pilot study were outlined below, taking into account some methodological considerations proposed by experts in the AAI field and trying to eliminate the basic weaknesses that sometimes arise in this type of project [29,30,31]. Furthermore, within the broad spectrum of animals used in AAE interventions, our intervention was specifically an experience of Dog Assisted Education.

### 2.4. Intervention

The intervention was an AAE program conducted by 2 AAI experts (one teacher and one psychologist). It was a goal-oriented, planned, and structured intervention focused on the development of psychomotor, social, cognitive, and communicative skills of children with special educational needs.

The AAE program was conducted twice a month for 45 min and took place at the Foundation’s facilities and also at the Rey Juan Carlos University Clinic, with each family choosing the setting according to the proximity to their home.

The therapeutic team was composed of 2 technical trainers and an AAI expert. The therapeutic staff had been involved in academic training either as an expert or as a technician (handler) in AAI, and their experience ranged from 3 to 12 years. The entire team was always present during the session.

The activities were designed trying to avoid the extenuation of participants. In turn, 14 sessions were planned, but 12 sessions were carried out for each group, as they had to be canceled due to the COVID19 pandemic. In any case, such a number of sessions is usually the most common number of sessions in this type of program [25,32].

Each session had a clear structure and timing, although slight adaptations or changes could be included in some sessions. The first part consisted of the greetings between the different members of the group. Then, the next part implied the feeding with different commands (seated, laid down, offering the paw, playing with canine cognitive toys) and the grooming. The third part was based on different games and physical activities with the dog. The activities of this part can be seen in Table 1. The fourth part included a relaxation period where contact with the dog was encouraged, working with the tactile sensations. Finally, the group said goodbye to the animals and professionals.

All sessions were designed to fulfill group and individual objectives, including group cohesion and interactions between members, as well as to specific and individual needs, which were consequences of their physical and/or intellectual disabilities.

### 2.5. Animal Wellbeing

The support dogs were of mixed breed. Both dogs were female, and their diverse basal activity rate was taken into account in order to participate in the activities [33]. These dogs followed strict zoonosis protocols, including behavioral, blood, urine, and feces analyses. Deworming, rabies, and tetravalent vaccinations were also required. Animal welfare was also ensured by the Animal Assisted Intervention Office of the University. A strict ethical protocol included some points to warrant animal welfare. First, the handler must live with the dog in the same house and, as pet and owner, they must have a strong mutual bond to ensure that the handler adequately identifies and interprets the emotions of the dogs during the intervention and avoid any kind of stress or fatigue. In this regard, all activities could be interrupted if the handler notes that the dog was not comfortable. Second, the selection of the dog and handler was based on the temperament, adaptability, motivation, and willingness to engage in the sessions. Third, the temperature of the facilities was always controlled to avoid very hot or very cold environments. Fourth, the dog was limited to 4 sessions/week with at least 20 min between-session rest in case there were 2 sessions on the same day. Furthermore, dog-training sessions must not exceed 4/day and must last less than 10 min each one. The dog must be fed before training in order to avoid hunger and must always have access to freshwater. Each session starts with a calm welcoming and ends with a calm goodbye. All this process was supervised by the head and academics of the Animal-Assisted Intervention Office of the University.

### 2.6. Statistical Analysis

Statistical analyses were performed using SPSS statistical software (IBM). Means and SD for each variable in each session were computed to describe the changes along the intervention. Linear mixed model analyses were conducted to analyze the influence of time and postural control on the rest of the indicators related to eye-motor coordination, language and communication, and sociability and autonomy.

Mixed models allowed us to estimate fixed effects and random effects. In our study, each individual was measured for the aforementioned indicators over 12 sessions. This was modeled by introducing a random constant in the mixed models analyzed. In this sense, linearity was assumed because it was understood that the effect of moving from session 1 to session 2 was the same as the effect of moving from session 2 to session 3. In turn, it was understood to be mixed because the observations of the variables were not independent but replicated of 14 individuals. The significance level chosen was as 0.05.

## 3. Results

Main characteristics of each participant are summarized in Table 2. A total of 10 boys and 4 girls were included in the study. All of them had in common the diagnosis of a disorder that caused physical and intellectual disability. The mean age was 88 months, and the SD was 35 months.

**Figure 1:**
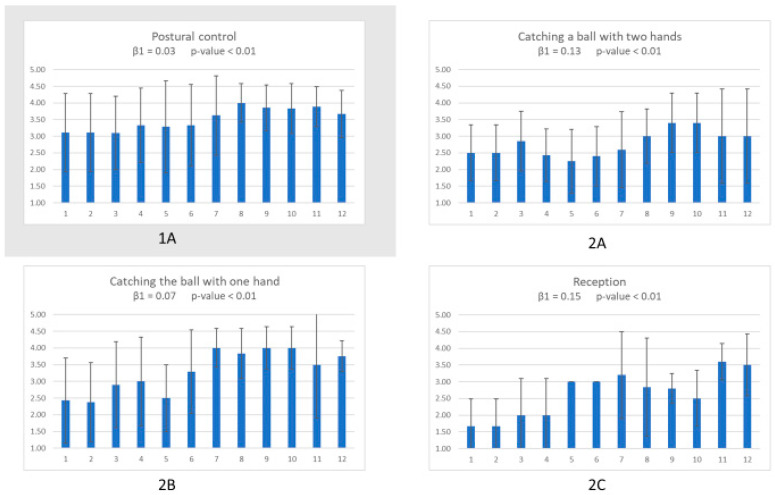
Fortnightly means and SD for postural control (**1A**) and the three eye-motor variables: catching and throwing a ball with two hands (**2A**), catching and throwing the ball with one single hand (**2B**), and reception of different objects (**2C**). Significant improvements were observed in all variables (*p* < 0.01).

Figure 1 shows the mean and SD of each postural control (1A) and eye-motor variables (2A–C) in each session. Mixed model results showed significant improvements in all these four variables (*p* < 0.01).

**Figure 2:**
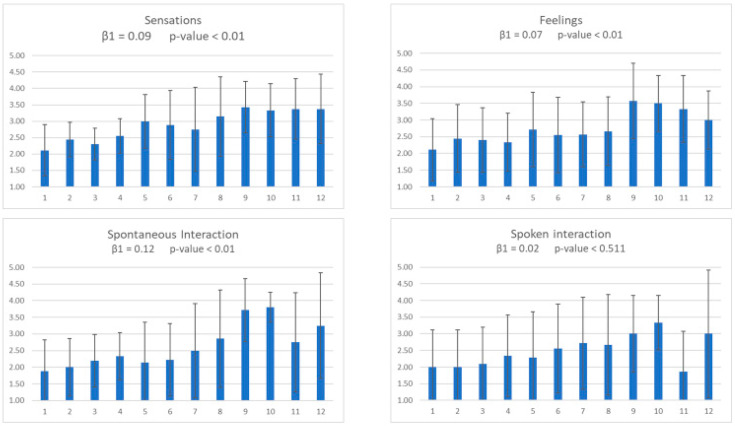
Fortnightly means and SD for the variable “Language and communication,” including expression of sensations, expression of feelings, spontaneous interaction, and spoken interaction. Significant enhancements were observed in all variables but spoken interaction.

Figure 2 shows the mean and SD of the items from the dimension “Language and communication.” These variables were the expression of sensations, expression of feelings, spontaneous interaction, and spoken interaction. Results showed a statistically significant improvement (*p* < 0.01) in all variables but spoken interaction (*p* = 0.511).

**Figure 3:**
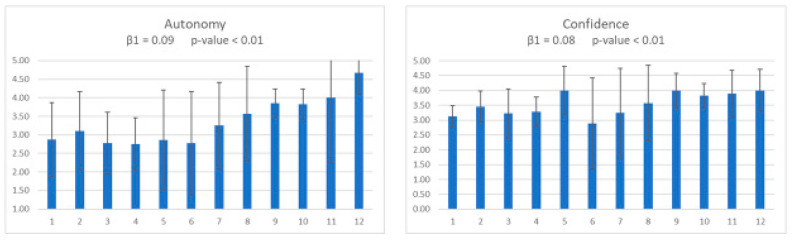
Fortnightly means and SD for the “autonomy” and “confidence” variables. Significant enhancements were observed in the two variables (*p* < 0.01).

The last two variables were autonomy and confidence. Results for these two variables are reported in Figure 3. Mixed model results showed a significant improvement for the two variables (*p* < 0.01).

## 4. Discussion

This pilot study aimed to evaluate the effects of a dog-assisted education program on the motor and communicative skills, as well as on the autonomy and confidence of children affected by different disorders that caused intellectual and physical disability. The findings of this pilot study indicated that, after the 12-session program, children who participated in the program improved their postural control, eye-motor coordination, expression of sensations and feelings, spontaneous interaction, autonomy, and confidence. These improvements could be extremely relevant for their development and daily living.

Although the current study is focused on the benefits in the children, it must be considered that every intervention involves a two-way relationship between the dog/s and the human/s. One relevant aspect of AAI is ensuring animal wellbeing. Previous studies reported that dogs could experience stress during AAI sessions [34,35], negatively impacting different aspects such as their immunological responses or leading to behavioral changes [36]. In this regard, dogs working in AAI should have the right of access to solid health, welfare, and wellbeing practices [37]. Our study is in line with the moral imperative of valuing dogs as sentient beings and, therefore, a strict ethical protocol was followed during all the process, from the selection and training of the dog to the interaction with the patients in the sessions. This protocol was controlled by the head and academics of the Animal-Assisted Intervention Office of the University. This protocol is not limited to the physical health of the animal through vaccines or blood, urine, and feces analyses, but also includes some points to ensure animal welfare, as can be read in the methods section. Previous studies have shown that dogs in well-planned and carefully designed AAT may be even positively affected when comparing their state before and after the session [38,39]. This was a priority in the current study, but animal welfare was only controlled through the observation of the handler and the Office staff but not systematically checked using objective, validated tools. Thus, future studies may include tools to objectively ensure animal wellbeing.

One of the main benefits of dog-assisted interventions is the improvement in social skills and communication, which is extremely useful in individuals with reduced social abilities, such as those with autism spectrum disorders [10]. The results of the current study are in line with those obtained by Becker, Rogers, and Burrows [32], who observed that, after 12 weeks of dog-assisted intervention, children aged 8-14 improved their social skills but not language development, which was the only single measure that was not significant in our study.

Although there were mixed results, AAI have shown improvements in the gross motor function, coordination, functional skills, or social interaction of children with cerebral palsy [26,40], which were in line with the results of the current study. However, it must be noted that AAT in this population usually involves horses instead of dogs [26,40]. One example of dog-assisted intervention in cerebral palsy was the study conducted by Elmacı and Cevizci [20]. They found that children with cerebral palsy learned to cope with their anxieties and fears, improving their abilities to use their bodies, their empathy, and their communication skills. Although there were some points in common, there were important differences between interventions with dogs and horses. First, hippotherapy involves the transmission of movement from the horse to the rider, while in dog-assisted interventions, the movement is facilitated by the dog but generated by the participant. Thus, the interaction with the horse is different, and the cause of the benefits may also be different compared to the interaction and the benefits from the dog-assisted interventions. One of the main advantages of dog-assisted interventions is the potential to be conducted in small, indoor or outdoor, urban or rural places, which enables the utilization in hospitals, nursing homes, prisons, etc.

The current pilot study supports the inclusion of AAI, and specifically dog-assisted programs, in the health system. A recent study showed that the possibility to include AAT in hospital may be accepted by hospital staff since they are aware of the benefits of these programs in children with disabilities such as cerebral palsy, autism spectrum disorder, or acquired brain injury [41]. The growing scientific literature on this field has shown that animals may positively influence human emotion and cognition [2]. Specifically, dogs have been identified as extremely helpful to elicit pro-social behavior and improve the communicative skills of children with disabilities, which is commonly a major problem [42,43,44].

Several limitations must be considered to adequately interpret the results of the current study. The first limitation is the lack of a control group, which prevents us from performing statistical analyses to observe between-group effects. The second limitation was the premature end of the program due to the COVID-19 pandemic, which meant the final two sessions were not conducted. The third limitation is the lack of standardized tests to evaluate the changes in the outcome measures as pre-post measures. This was decided by the researchers along with the families and the staff of the foundation in order to minimize the evaluations in children who are frequently undergoing medical tests. Future research must confirm the results reported in the current pilot study using standardized measures. The last fourth limitation was the low sample size and the heterogeneity in the diagnosis and characteristics of participants. Although all 14 participants were children affected by physical and intellectual disabilities, there were relevant differences in functionality and development. Furthermore, the lack of a systematic tool to evaluate the effect of the therapy in the dog may also be considered as another limitation. Despite all these limitations, the current pilot study provides evidence of the benefits of a dog-assisted intervention in a sample comprised of children with different types of physical and intellectual disabilities.

## 5. Conclusions

A total of 12 sessions of dog-assisted education led to improvements in postural control, eye-motor coordination, communication, autonomy, and confidence in children with different types of physical and intellectual disabilities. The findings of this study protocol support the inclusion of AAI in the health system but must be interpreted with caution due to the lack of a control group, the sample size, and the heterogeneity of the sample.

## Figures and Tables

**Table 1:** Example of activities included in the dog-assisted education program “One dog, one happy child”.

Activity	Description
Walking the dog	The activity consisted of handling a dog and drive it through a circuit with pikes and hops.
Catch the ball	The activity implies the launch of balls with different textures and colors in order to follow the instructions in each trial.
Color’s island	The child must guide the dog to the cone colored with the same color that the card extracted by him/her. It is convenient to encourage the group to help the child during the task.
Game of tissues	This activity consisted of using two colored tissues (green and red) and, depending on the cloth raised by the technician, move along with the dog through the classroom (green) or freeze and stay quiet (red). Attention and social functions are activated with this task.
Game of emotions	The emotions expressed by dogs must be recognized by the children using stuck cards with different faces.

**Table 2:** Main descriptive characteristics of the participants.

Participant	Sex	Age (Months)	Condition
1	M	55	Transposition of the great vessels.
2	M	120	Mowat-Wilson syndrome
3	M	91	Kabuki syndrome
4	F	150	FoxG1 syndrome
5	M	137	Peripheral neuropathy
6	M	54	Stroke
7	M	75	Cerebral Palsy
8	M	67	Kallman syndrome
9	F	78	Cerebral Palsy
10	F	46	Cerebellar hypoplasia
11	M	120	Angelman syndrome
12	M	122	Congenital disorders of glycosylation
13	F	61	Propionic acidemia
14	M	56	Physical and intellectual disabilities but diagnosis not provided by his parents

## Data Availability

Data would be available upon reasonable request to corresponding author.
